# Time-Dependent Kinetic Complexities in Enzyme Assays: A Review

**DOI:** 10.3390/biom15050641

**Published:** 2025-04-30

**Authors:** Juan Luis Rendón, Juan Pablo Pardo

**Affiliations:** Departamento de Bioquímica, Facultad de Medicina, Universidad Nacional Autónoma de Mexico, Apartado Postal 70-159, Ciudad de México 04510, Mexico; pardov@bq.unam.mx

**Keywords:** kinetic complexities, hysteresis, enzyme assay, burst, lag

## Abstract

In the present review, the importance of analyzing full progress curves in enzyme assays is discussed. The atypical kinetic behavior that can be potentially displayed by enzymes in the performance of an activity assay, as well as the models explaining such behavior, are analyzed. These complex time-dependent kinetic patterns include hysteresis, damped oscillatory hysteresis, unstable product, and kinetic competence. The atypical time-dependent patterns are discussed with both real examples and In Silico simulations. When possible, the physiological implications of such kinetic behaviors are included. The importance of analyzing the derivative of the reaction rate of such atypical transitions as a method to distinguish them from the conventional non-atypical time progress curve is stressed.

## 1. Introduction

The determination of the catalytic activity of an enzyme is essential for its full characterization. Although the specific protocol designed for a particular enzyme will depend on a variety of factors, in an ideal scenario, it is necessary to have a method that allows for continuous monitoring of either the disappearance of a substrate or the formation of a product (e.g., in the form of an absorbance versus time plot). From such a record, the initial velocity (i.e., the initial slope) of the reaction under a particular set of conditions can be evaluated [1,2,3]. The initial velocity of an enzyme-catalyzed reaction serves as the foundation for the determination of the basic kinetic parameters (e.g., *K_m_* and *k_cat_*) of an enzyme [1,2,3]. Further, the application of the mathematical framework of enzyme kinetics—either from the steady-state or the rapid equilibrium approaches—relies on the experimental determination of initial velocities. While it is also possible to estimate the kinetic parameters from the full progress curve of an enzyme-catalyzed reaction [4,5], this approach has been limited in use. In this latter approach, an experimental progress curve is compared with that generated through numerical integration of a set of differential equations obtained from a model-based mechanism [4]. Alternatively, experimental data (e.g., the absorbance or fluorescence signal as a function of time) are fitted by non-linear regression to an empirical function that includes both *K_m_* and *V_m_* [6,7,8]. Regardless of the method used for the analysis, the conventional profile of the full progress curve of an enzyme catalyzed reaction is characterized by a continuous decrease in the concentration of a substrate (or a continuous increase in product concentration), such that the velocity (i.e., the derivative of substrate concentration with regard to time) is highest in the early stage of the reaction and decreases continuously. Such a decrease will be the result of a variety of factors, such as a decline in substrate concentration and/or the accumulation of a potentially inhibitory product. For a reversible reaction, the effect of the reverse reaction will also be an important factor in the decrease in the reaction velocity.

While the initial velocity of an enzyme-catalyzed reaction often provides reliable insights into the enzyme’s kinetic behavior, there are instances in which relying solely on this variable can lead to erroneous conclusions. In such cases, the careful analysis of the full progress curve of the reaction may yield additional useful information. The aim of the present review is to discuss the atypical kinetic behavior displayed by various enzymes in the activity assays, as well as the underlying molecular basis. This topic is largely overlooked in standard enzymology textbooks [9]. The work will be particularly beneficial for researchers who may lack experience in identifying and understanding the potential deviations from the expected conventional enzyme behavior. Although some enzyme inhibitors (e.g., slow-binding inhibitors) also display a time-dependent atypical kinetic behavior in an enzyme assay, these are not covered in the present review. Excellent works covering this topic are available [10,11,12].

### Theoretical Background

**Scheme 1:**
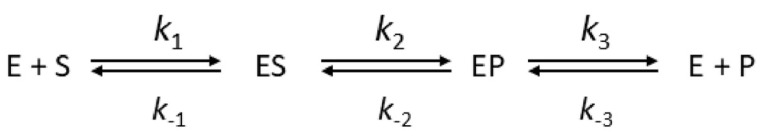
Kinetic model for a reversible uni–uni catalyzed enzyme reaction.

**Figure 1:**
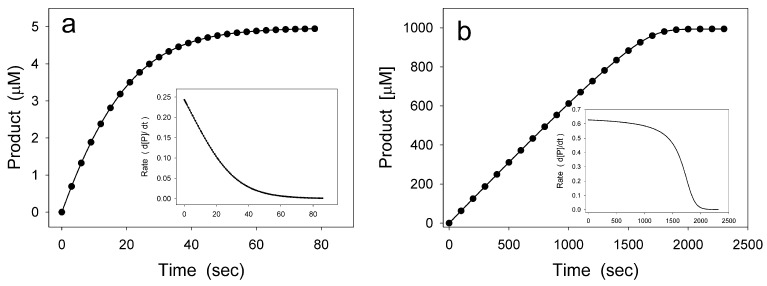
In Silico simulations of full progress curves for a conventional uni–uni enzyme-catalyzed reaction. Plots were generated by Dynafit software (version 4) [18] based on the model shown in Scheme 1. The corresponding rate constants values used were as follows: *k*_1_ = 25 µM^−1^ s^−1^; *k*_−1_ = 180 s^−1^; *k*_2_ = 90 s^−1^; *k*_−2_ = 20 s^−1^; *k*_3_ = 260 s^−1^; *k*_−3_ = 1 µM^−1^ s^−1^. Data were simulated either at a low (5 µM) (**a**) or high (1 mM) (**b**) substrate concentration. For both conditions, an enzyme concentration of 10 nM was used. Insets show the corresponding variation in the instantaneous reaction rate as a function of time.

In the earliest stages of an enzyme-catalyzed reaction—the so-called pre-steady state stage—the formation of the enzyme–substrate Michaelian complexes occurs. Such a stage occurs in a very short time, typically in the range of micro- to milliseconds, and results in the quick appearance of both the transitory and the central complexes involved in the catalysis [13,14]. For a simple reversible uni–uni reaction, such as that shown in Scheme 1, the observed initial velocity will depend on the magnitudes of both the first and second order specific rate constants. In the rapid equilibrium approach, it is assumed that the rate at which the E + S ↔ ES reaches equilibrium is faster compared to both the isomerization of the central complexes ES and EP and the release of the product(s). Such differences in velocities will be determined by the magnitudes of the rate constants involved (under such conditions, the *K_m_* can be considered as an authentic dissociation constant). In contrast, under steady-state conditions, the values of all the rate constants involved are in a similar range. For both cases, however, the experimental determination of the numerical value for a particular rate constant requires the use of fast reaction techniques [15,16,17]. Additionally, the rate in a full progress curve depends on the specific concentrations of both the substrate and the enzyme used in the activity assay. At low substrate concentrations, a significant decrease in the slope of the trace is observed due to a rapid decrease in the concentration of both the ES and EP complexes (inset of Figure 1a). Conversely, at a very high substrate concentration (over 50 times the *K_m_*), the steady-state concentration of the productive complexes of the enzyme will remain constant for longer times, and significant changes in the velocity of the reaction will be evident only until the final stage of the reaction (inset of Figure 1b). Nevertheless, in both cases, the slope is maximal at the start of the reaction.

However, for some enzymes, the typical kinetic behavior described above does not hold. In such cases, the reaction velocity will vary in an unexpected fashion, resulting in an atypical full progress curve. Albeit, in some cases, the atypical behavior will be evident from a simple visual inspection of the kinetic trace, there are situations in which a more careful analysis will be needed. In such cases, the first derivative of the full progress curve will be of great utility. A more detailed analysis will require a knowledge of the kinetic mechanism followed by the enzyme to set up a plausible model that can explain the atypical kinetic profile.

## 2. Hysteretic Enzymes

**Figure 2:**
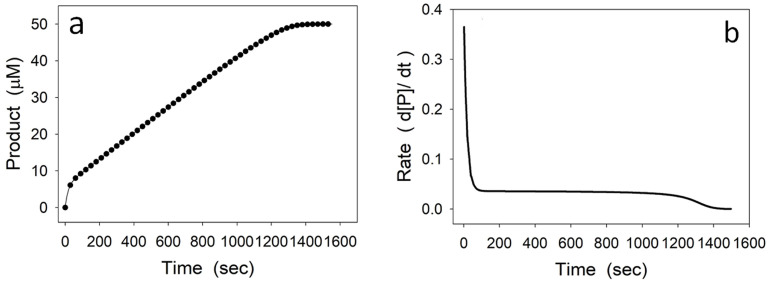
In Silico simulation of a time progress curve for a burst hysteretic transition (**a**). Plots were generated by Dynafit software (version 4) [18] based on the model shown in Scheme 2. The corresponding rate constants values used in the simulation were as follows: *k*_1_ = 0.145 s^−1^; *k*_−1_ = 0.0015 s^−1^; *k*_2_ = 20 µM^−1^ s^−1^; *k*_−2_ = 350 s^−1^; *k*_3_ = 80 µM^−1^ s^−1^; *k*_−3_ = 90 s^−1^; *k*_4_ = 0.4 s^−1^; *k*_−4_ = 0.008 s^−1^; *k*_5_ = 250 s^−1^; *k*_5_ = 15 s^−1^. Data were simulated at 50 µM of substrate concentration, and an enzyme concentration of 2.5 nM was used. Panel (**b**) shows the corresponding variation in the instantaneous reaction rate as a function of time.

**Figure 3:**
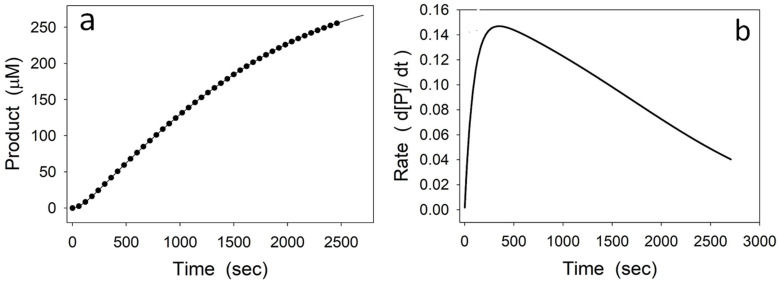
In Silico simulation of a time progress curve for a lag hysteretic transition (**a**). Plots were generated by Dynafit software (version 4) [18] based on the model shown in Scheme 2. The corresponding rate constants values used in the simulation were as follows: *k*_1_ = 0.0015 s^−1^; *k*_−1_ = 0.015 s^−1^; *k*_2_ = 10 µM^−1^ s^−1^; *k*_−2_ = 120 s^−1^; *k*_3_ = 1 µM^−1^ s^−1^; *k*_−3_ = 40 s^−1^; *k*_4_ = 0.001 s^−1^; *k*_−4_ = 0.008 s^−1^; *k*_5_ = 80 s^−1^; *k*_6_ = 0.1 s^−1^. Data were simulated at 80 µM of substrate concentration, and an enzyme concentration of 30 nM was used. Panel (**b**) shows the corresponding variation in the instantaneous reaction rate as a function of time.

**Scheme 2:**
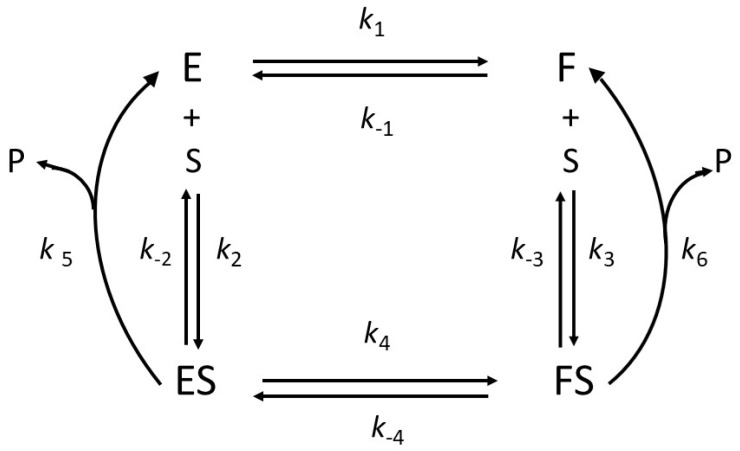
General kinetic model for a reversible uni–uni enzyme-catalyzed reaction showing a hysteretic behavior.

The concept of hysteresis in enzyme kinetics was introduced in 1970 by Carl Frieden [19] for enzymes that exhibit a slow response to a sudden change in ligand concentration, typically a substrate. Many enzymes showing hysteresis have been described [20], representing the major group of enzymes with atypical progress curves. This behavior is characterized by an initial velocity that does not match the true steady state velocity, which is reached later in the reaction. The transition between the two velocities is a critical point explaining the behavior of these enzymes, which is characterized by a slow process. During this transition, the reaction rate gradually changes from an initial value (*Vi*) into the final steady state velocity (*Vss*) due to a continuous change in the relative abundance of two enzyme species (Scheme 2) [19,20]. Based on the relationship between the two velocities, two types of hysteretic responses are recognized: (a) enzymes showing a lag in enzyme activity [21,22,23,24,25,26,27,28,29,30,31,32,33,34,35,36], such that the initial velocity is lower than the steady-state velocity (in an extreme situation, the initial velocity is zero); and (b) enzymes showing a burst of activity in the progress curve [37,38,39,40], where the initial velocity is higher than the steady state velocity. Interestingly, there are also reports on enzymes showing both burst and lag hysteretic behavior [35,41,42]. The kinetic behavior of both kinds of hysteretic enzymes is illustrated in Figure 2 (burst) and Figure 3 (lag), showing the full-time course of product formation. The activity profiles are essentially the same when substrate consumption is followed. For the latter situation, activity traces start at a non-zero concentration and display negative slopes throughout the time course.

At first sight, the full progress curves for hysteretic enzymes seem similar when compared with the profile of a conventional non-hysteretic enzyme (Figure 1). However, when the initial stages of the reactions are contrasted, the differences become clear. Figure 5 and Figure 6 show the profile of the initial stages of the reaction for both a burst- or a lag-hysteretic transition.

**Figure 4:**
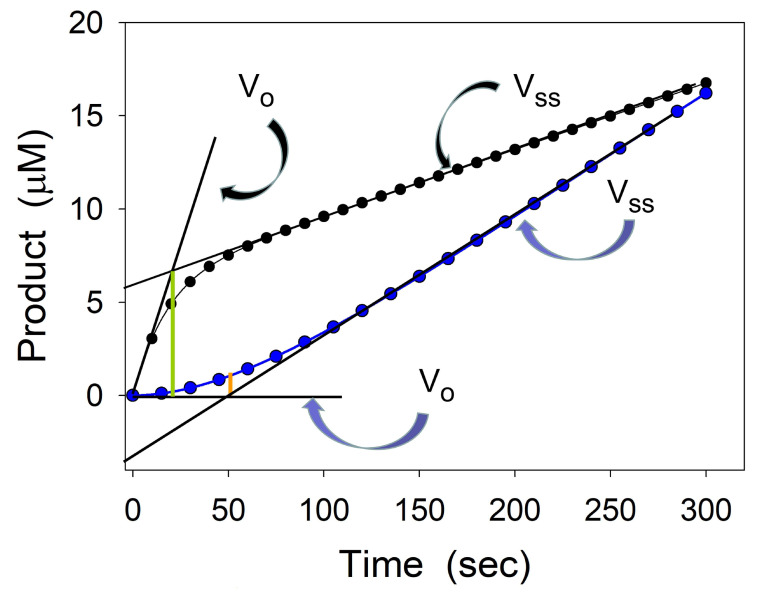
In Silico simulation of the initial stages for a reaction catalyzed by an enzyme showing either a burst (black) or a lag (blue) hysteretic transition. Plots were generated by Dynafit software (version 4) [18] based on the model shown in Scheme 2. The corresponding rate constant values used in the simulations were as indicated in the legend to Figure 4 (burst) or Figure 5 (lag). Substrate concentration used in the simulations was 50 µM for the burst transition or 80 µM for the lag transition. The intersection point with the “*y*” axis of the extrapolated lines represents the excess (burst) or deficit (lag) in product concentration. Both initial and steady-state velocities, as well as the lag time for each hysteretic transition, are indicated. The corresponding lag time for each transition is shown as vertical green (burst) or orange (lag) lines (see text for details).

From the initial stages of either a burst or a lag hysteretic transition (Figure 4), the following information can be obtained:

The initial velocity (*V_i_*): This experimental data represents the activity of the enzyme species present in the reaction mixture at the start of the reaction, before the transition involved in the atypical progress curve takes place. For hysteretic enzymes with a lag in the progress curve, the slope of the kinetic trace will continuously increase from an initial value up to the steady-state velocity. In the case of an inactive initial enzyme form, the initial velocity is zero. This last situation must be emphasized because the observation of a zero initial slope could lead the worker to the erroneous conclusion that the enzyme under study is absent or dead in the reaction mixture. Following the reaction for longer times could reveal the presence of catalytic activity. On the other hand, for hysteretic enzymes showing a burst in their activity profile, the slope of the progress curve will continuously decrease up to a linear segment corresponding to the steady-state velocity.

The steady state velocity (*V_ss_*) corresponds to the activity of the enzyme form present at the end of the transition. This velocity can be estimated from the linear segment of the full progress curve. Unlike the initial velocity, however, the experimental determination of the steady-state velocity for a hysteretic enzyme is by no means easy because the presence of a segment of constant slope in the progress curve is frequently not evident, particularly at low substrate concentrations. In this situation, it will be necessary to evaluate the first derivative of the experimental data to determine the point of maximal slope.

The specific rate constant (*k*): This parameter is related to the slow transition between the enzyme forms involved in the atypical progress curve. It can be calculated directly from the full progress curve of the reaction by obtaining the lag time from the intersection point of the slopes corresponding to the initial and the steady-state velocities, as shown in Figure 4. The specific rate constant is the reciprocal of the lag time, and its magnitude depends on all the rate constants involved in the model [20,43] as well as the substrate concentration. For many hysteretic enzymes, the lag time is in the range of seconds to minutes [21,22,23,24,25,26,27,28,29,30,31,32,33,34,35,36,37,38,39,40].

The amplitude of the transition represents either the excess (for enzymes showing a burst in activity) or the deficit (for enzymes with a lag in their activity profile) in product formation. This parameter is obtained by extrapolating the line defining the steady-state velocity to its intersection with the concentration axis (see Figure 4).

By using product formation to follow the progress of the reaction, the above-defined parameters are related through the equation:

(1) $$[P]=Vsst-(Vss-Vi)(1-e^{-kt})/k$$

where *P* stands for product concentration as a function of time *t*. The equation is defined for kinetic traces in which a lag in activity is present (a detailed derivation of Equation (1) can be found at the end of reference [10]). For enzymes showing a burst in their full progress curve, the term corresponding to the difference in velocities must be replaced by (*Vi* − *Vss*). When the steady state condition is reached (i.e., the slope of the progress curve becomes constant), Equation (1) simplifies to the following:

(2) [P]=Vsst−(Vss−Vi)/k

where [*P*] stands for product concentration during the steady-state velocity segment of the reaction. This condition is reached only at high substrate concentrations. By deriving Equation (1), a function defining the instantaneous reaction velocity (*V*) along the progress curve is obtained:

(3) $$V=\frac{d[p]}{dt}=Vss-(Vss-Vi)e^{-kt}$$

At present, various computer programs are available to fit experimental data from full progress curves to the previously defined equations [6,7,18].

**Figure 5:**
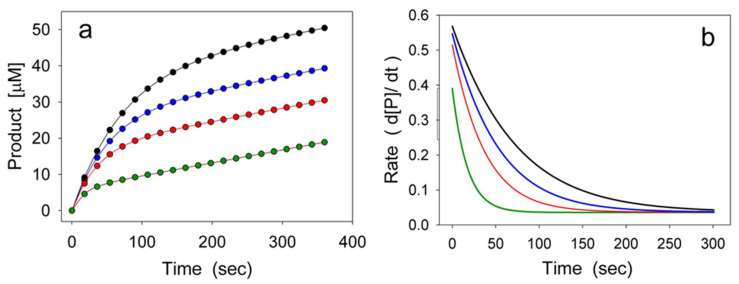
In Silico simulations of the initial stages of the reaction for a burst hysteretic transition. (**a**) Effect of substrate concentration on the profile of progress curves. Data were simulated with Dynafit software (version 4) [18] based on the model shown in Scheme 2, assuming “E” as the most active species. (**b**) Instantaneous reaction rates as a function of time. The rate constant values used were as in Figure 2. Substrate concentrations (in the micromolar scale) were as follows: 50 (green line); 140 (red line); 210 (blue line); 300 (black line). The initial concentration of the enzyme species “E” was 2.5 nM.

**Figure 6:**
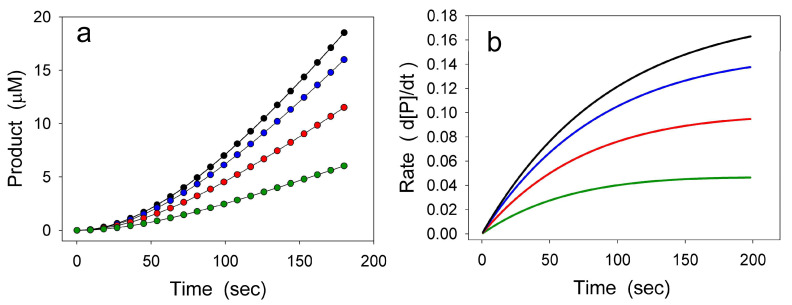
In Silico simulations of the initial stages of the reaction for a lag hysteretic transition. (**a**) Effect of substrate concentration on the profile of progress curves. Data were simulated with Dynafit software (version 4) [18] based on the model shown in Scheme 2, assuming “F” as the most active species. (**b**) Instantaneous reaction rates are shown in panel (**b**). The rate constant values used in the simulation were as in Figure 3. Substrate concentrations (in the micromolar scale) were as follows: 50 (green line); 140 (red line); 300 (blue line); 500 (black line). The initial concentration of the enzyme species “E” was 30 nM.

In Silico simulated progress curves representing the initial stages of the reaction for the two hysteretic patterns, as well as their corresponding derivative plots, are shown in Figure 5 and Figure 6. For each curve, only the segment representing the attainment of the steady-state velocity is shown. These curves were obtained at different substrate concentrations to illustrate the effect of this variable both on the profile of the progress curve (Figure 5a and Figure 6a) as well as the variation in the instantaneous reaction rate (Figure 5b and Figure 6b). Comparing these profiles with that of a conventional non-hysteretic enzyme (Figure 1), particularly with the profile of the instantaneous rates, will clarify the differences between the two types of kinetic behavior.

### 2.1. General Model of Hysteretic Behavior

To explain the molecular basis of the kinetic behavior of hysteretic enzymes, a simple uni–uni reaction catalyzed by an enzyme will be used as a model (Scheme 2). The assumptions of the model are as follows:

In the absence of a ligand, the enzyme population consists of two alternate states, the active and inactive (or less active) forms of the enzyme. Both states coexist in a slow equilibrium, with the rate constants characterizing this equilibrium being small compared to the rate constant for the conversion of substrate into product (i.e., *k*_1_ + *k*_−1_ << *k*_6_), thus representing the rate-limiting step. Alternatively, the enzyme may exist in a single state, but the addition of the substrate allows it to access a new state. In this situation, the transition must also be slow to observe a hysteretic transition.

The kinetic properties of the two states are different. This is the most critical feature of the model, allowing the possibility to detect an atypical full progress curve in the enzyme assay. In the kinetic model shown in Scheme 2, it is assumed that both the E and F species can bind substrate, and the resultant ES and FS enzyme–substrate complexes are active, although with different catalytic efficiency. Hence, the observation of a lag or a burst during the progress curve will depend on the relative activities (defined by the catalytic constants *k*_5_ and *k*_6_) of the “F” and “E” states, as well as their relative abundance in the enzyme population. Assuming “F” as the more active species, with a higher affinity for the substrate as compared with “E”, and representing a minor fraction in the initial population of the enzyme, the addition of the ligand will result in a lag in the progress curve. On the contrary, assuming “E” is the more active form of the enzyme, and under the same initial conditions, then a ligand favoring the accumulation of “F” by shifting the equilibrium toward the right will allow the observation of a burst.

### 2.2. Molecular Origin of Hysteretic Behavior

While the model depicted in Scheme 2 does not provide information about the differences in molecular structure between the “E” and “F” states of the enzyme that explain its atypical kinetic behavior, experimental results from various enzymes exhibiting hysteretic behavior [21,22,23,24,25,26,27,28,29,30,31,32,33,34,35,36,37,38,39,40] have offered insight into the molecular basis of such behavior. These can be summarized as follows:

(i) A slow conformational change: The addition of the ligand involved in the hysteretic phenomenon will displace the conformational equilibrium toward either the “ES” or “FS” complexes. Ligand binding occurs under rapid equilibrium conditions, so the observed transition in the activity assay will be the result of the slow conformational transition. Such structural transitions can be directly detected by following changes in the intrinsic fluorescence of the protein [44]. Examples of hysteretic enzymes showing a slow conformational change include pyruvate carboxylase [42], fructose 1,6-bisphosphatase [29], ribulose 1,5-bisphosphate carboxylase [45], methane monooxygenase [46], and 5-aminolevulinate synthase [47].
(ii) A change in the aggregation state of the enzyme: In this case, binding of the ligand involved in hysteretic behavior will result in either dissociation or aggregation of the enzyme. For protein aggregation, the velocity of the transition is strongly influenced by protein concentration in the enzyme assay. Like the conformational changes, ligand binding occurs under rapid equilibrium conditions. When the change in the aggregation state of the enzyme is very slow, on the scale of several minutes, the transition can be analyzed using a combination of covalent cross-linkers and denaturing electrophoresis [48]. Examples of enzymes showing changes in their aggregation states, concomitant with hysteretic kinetics, include phosphofructokinase [49], CTP synthetase [50], glutamine phosphoribosylpyrophosphate amidotransferase [30], malic enzyme [51], and hexokinase [52].
(iii) Displacement of a tightly bound ligand: In this model of hysteretic behavior, a compound with a very small dissociation constant, in the range of 10^−7^ M or less, is displaced from the enzyme in the presence of an alternative ligand, typically the enzyme substrate. As a result, the progress curve of reaction shows a gradual increase in velocity. Glutamate dehydrogenase from bovine liver displays this kind of behavior with GTP as an inhibitor [19].
(iv) Hysteretic transitions can also arise from sudden changes in either pH [31,53,54] or temperature [32,55]. Such transitions may potentially be observed when the enzyme assay conditions differ from those of its storage [56]. The underlying molecular cause can be attributed to either conformational or aggregation changes. In the case of sudden changes in pH, differences in the protonation state of ionizable groups are the origin of the structural changes.
(v) Hysteresis by substrate channeling: In some Gram-negative bacteria, such as *Escherichia coli* and *Helicobacter pylori*, proline catabolism is dependent on a bifunctional flavoenzyme named as proline utilization A (PutA) [21,57]. The enzyme catalyzes the NAD-dependent oxidative conversion of proline into glutamate through the activity of two physically separated but functionally coupled active sites: proline dehydrogenase (PRODH) and glutamate semialdehyde dehydrogenase (GSAL) [21]. The corresponding reactions are as follows:
  $$\mathrm{Proline}+\mathrm{Ubiquinone}\;\overset{PRODH}{\to }\;{\text{∆}}^{1}\text{-}\mathrm{Pyrroline}\text{-}5\text{-}\mathrm{carboxylate}+\mathrm{Ubiquinone}(H_{2})$$
  $${\text{∆}}^{1}\text{-}\mathrm{Pyrroline}\text{-}5\text{-}\mathrm{carboxylate}+H_{2}O\;\overset{Spontaneous}{\to }\;L\text{-}\gamma \text{-}\mathrm{semialdehyde}$$
  $$L\text{-}\mathrm{glutamate}\text{-}\gamma \text{-}\mathrm{semialdehyde}+H_{2}O+{\mathrm{NAD}}^{+}\;\overset{GSAL}{\to }\;\mathrm{Glutamate}\;+\;\mathrm{NADH}\;+\;H^{+}$$

The reaction product of PRODH (∆^1^-pyrroline-5-carboxylate) undergoes a non-enzymatic hydrolysis into L-glutamate-γ-semialdehyde, which is converted into glutamate by GSAL. The corresponding active sites are in distinct domains of the polypeptide chain [58], and, hence, a channeling mechanism is necessary to transport the reaction intermediary between the active sites. Kinetic studies of the *E. coli* enzyme have revealed the existence of a lag-type hysteretic transition, which is proposed to be due to the channeling event [21]. In this model, the microscopic rate constant for the coupled reaction increases continuously with subsequent turnovers, reaching the final steady-state kcat value with subsequent turnovers of the enzyme, resulting in hysteresis [59]. Interestingly, *H. pylori* colonize the upper gastrointestinal tract where proline concentration is high [60], thus supporting this bifunctional enzyme as a potential drug target.

While it has been noted that the ligand binding involved in the hysteretic transition occurs under rapid equilibrium conditions, this is not a necessary requirement. It is possible that the rate constants for ligand binding and dissociation are in the same order of magnitude as compared to those associated with the slow transition of the enzyme. In this case, the hysteretic phenomenon will be accompanied by kinetic cooperativity, which could be detected by analyzing the dependence of the initial velocity on ligand concentration [43]. Either positive or negative cooperativity may be observed, associated with lag or burst transitions [61].

### 2.3. Hysteretic-like Progress Curves

The observation of either a lag or a burst in an enzyme assay could, in principle, suggest the presence of hysteretic behavior. However, researchers must be aware of the existence of a variety of situations in which non-hysteretic full progress curves could be detected. The most common causes are discussed briefly:

In those cases where an auxiliary enzyme is present, such as in coupled enzyme assays, a lag would be observed when the concentration of the auxiliary enzyme is not high enough [62]. By increasing the concentration of the latter, the slow transition must be eliminated. For the case of an authentic hysteretic enzyme requiring an auxiliary enzyme, a method to analyze its kinetics is available [63].

The presence of a slow-binding inhibitor in the enzyme assay will result in the observation of a burst-like transition. In this setting, the slow formation of the enzyme-inhibitor complex is in the origin of the atypical transition [10].

The time-dependent inactivation of the enzyme during the progress of the enzyme-catalyzed reaction could similarly result in a burst-like transition. To discard this possibility, additional experiments in which the enzyme is preincubated under the conditions of the enzyme assay at various times would be needed. A decrease in the initial velocity will reveal the presence of an inactivation phenomenon, thus discarding a hysteretic transition.

The slow covalent modification of specific residues on an enzyme could also result in the observation of a lag-type profile in the time progress curves. This situation is exemplified by phosphorylase kinase (PhK), an important regulatory enzyme in glycogen metabolism [64]. The activity of PhK is critically dependent on the Ca^++^ and Mg^++^-dependent phosphorylation of several serine residues of its β subunits [33]. In the experimental progress curves of PhK activity, a pronounced lag is observed [65]; however, by preincubation in the presence of both Ca^++^ and Mg^++^, such lag period is fully abolished [33].

### 2.4. Is It Possible to Avoid Hysteretic Behavior in an Enzyme Activity Assay?

Although the hysteretic phenomenon is an intrinsic property of a particular enzyme, which is only revealed in an activity assay under specific conditions, and is essential in its characterization, it is feasible to avoid its manifestation in certain circumstances. For monomeric enzymes, in which either the substrate or a cofactor (if necessary) is involved in the hysteretic response, preincubation of the enzyme in its presence can eliminate the atypical profile of the progress curve. The required preincubation time will depend on the magnitude of the lag time (i.e., seconds or minutes) and must be determined experimentally.

On the other hand, in those cases where more than one substrate is involved in the reaction, the possibility of avoiding the hysteretic response will be strongly dependent on the kinetic mechanism followed by the enzyme. Thus, when the order of addition of the substrates is under random conditions, preincubation with the substrate involved in the hysteresis will allow us to avoid the phenomenon. However, for sequential kinetic mechanisms (e.g., ping pong or ordered), the elimination of the lag time will be possible only when the first substrate in the sequence is responsible for the atypical behavior. This last situation stresses the importance of understanding the kinetic mechanism of the enzyme.

## 3. Physiological Significance of Hysteretic Behavior

The occurrence of enzymes with atypical kinetic transitions encouraged interest in their potential physiological significance. In this section, some hysteretic enzymes, as well as their putative physiological significance, are briefly discussed.

### 3.1. Hysteresis in Metabolic Pathways

**Scheme 3:**
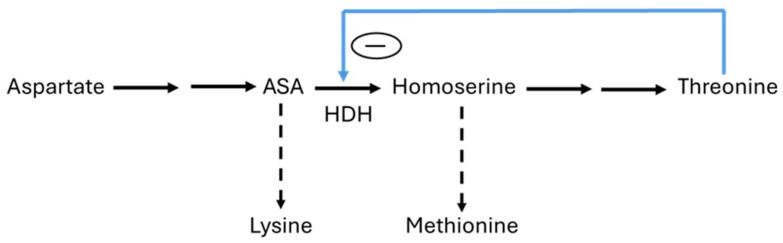
Simplified threonine biosynthesis pathway in *E. coli*. Solid black arrows represent an enzyme-catalyzed reaction. Blue arrow represents feedback inhibition by threonine; dashed lines represent a metabolic branch leading to either Lysine or methionine. HDH:Homoserine dehydrogenase.

In *Escherichia coli*, the metabolism of the amino acids aspartate, threonine, lysine, and methionine is tightly coupled, as shown in Scheme 3 [66]:

In this metabolic branched pathway, the final product threonine acts as a feedback regulator through its inhibition of both homoserine dehydrogenase and homoserine kinase [66]. Thus, when threonine availability in the culture medium is high, the rate of the reactions leading to its production will be decreased. However, due to the importance of homoserine for methionine synthesis, the production of the latter will also be affected by the high threonine levels. In this sense, it is interesting to note that the response of homoserine dehydrogenase to threonine is slow, showing a time delay in its inhibition [67]. Under such conditions, this hysteretic response of homoserine dehydrogenase to threonine will allow bacteria to buffer the methionine synthesis against changes in threonine levels.

A second example of the importance of hysteretic enzymes in metabolic pathways is represented by Phenylalanine hydroxylase (PAH) [Phenylalanine 4-monooxygenase EC 1.14.16.1]. This enzyme belongs to the mixed function oxidases family and catalyzes the first and rate-limiting step in phenylalanine catabolism according to the reaction:

Phenylalanine+O2+Tetrahydrobiopterin ⟶................ Tyrosine+Dihydrobiopterin+H2O

**Figure 7:**
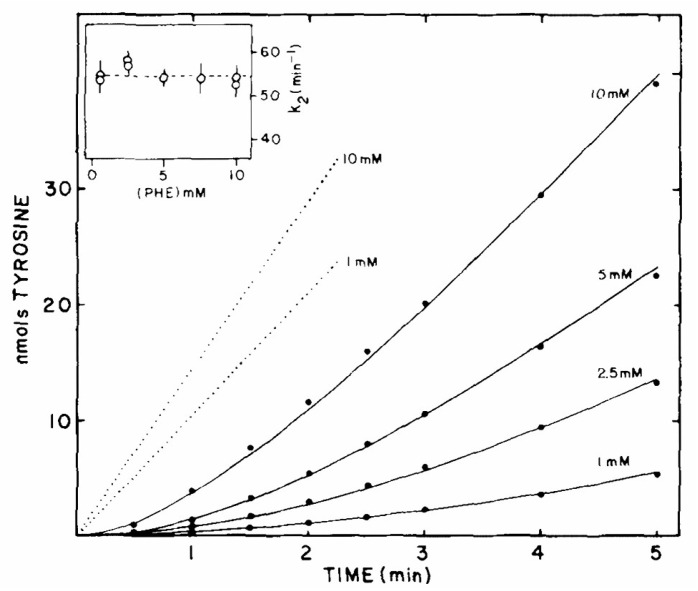
Experimental progress curves showing the initial stages of the reaction catalyzed by phenylalanine hydroxylase from rat liver at various concentrations of phenylalanine. Reactions were carried out at 11.5 °C in 0.1 M potassium phosphate buffer (pH 6.8) containing 60 µM 6-methyltetrahydrobiopterin and 6 mM dithiothreitol. Solid lines represent reactions started by the addition of 5 µg of enzyme. Dashed lines correspond to reactions in which the enzyme was preincubated for 3 min with phenylalanine and the reaction started by adding methyltetrahydrobiopterin. Numbers indicate the final concentration (in the millimolar scale) of phenylalanine in the reaction mixture. Figure and data were taken from reference [72] with permission.

The enzyme has a dual physiological role: it participates in the endogenous synthesis of tyrosine and catalyzes the first step in phenylalanine degradation when the amino acid is in excess [68]. A deficiency in the activity levels of PAH occurs in the origin of phenylketonuria, the most prevalent congenital metabolic disorder [68]. The enzyme is a tetramer of identical subunits [69,70], and each monomer is organized into an N-terminal regulatory domain, a large central catalytic domain, and a C-terminal multimerization domain [70,71]. The kinetic behavior of PAH is complex, showing both allostery and hysteresis. By varying phenylalanine concentration, the enzyme displays positive cooperation. Interestingly, in the activity assays of PAH, a clear lag period is observed when the reaction is started by the addition of phenylalanine (Figure 7) [72], revealing a time-dependent activation of the enzyme. This lag is abolished by preincubation of the enzyme with the aromatic amino acid. Based on detailed structural studies of the enzyme, it has been possible to reveal a second binding site for phenylalanine at the N-terminal regulatory domain of the enzyme [69,73]. A model intended to explain the hysteretic behavior of PAH [70] has been proposed, in which the enzyme exists as an equilibrium mixture between inactive and active tetramers, which are conformationally distinct. The transition between the two oligomers is slow, and phenylalanine stabilizes the active tetramer, thus explaining the lag observed in the enzyme assays.

The hysteretic behavior of PAH can be viewed as a physiological strategy for regulating enzyme activity in response to fluctuations in phenylalanine availability. Thus, under limited supply conditions of the amino acid, the low activity of PAH will allow phenylalanine to participate in protein biosynthesis. In contrast, when phenylalanine is in excess, the faster activation of the enzyme by the amino acid will divert the flux toward the conversion of phenylalanine to tyrosine for catabolic purposes.

### 3.2. Hysteresis as an Adaptation to Environmental Changes

In the brine shrimp *Artemia*, trehalose is the primary fuel in the early embryogenesis [74], and hence, the hydrolytic activity of trehalase (α,α-trehalose glucohydrolase EC 3.2.1.28) is critical for glucose availability. Depending on environmental and physiological conditions, *Artemia* embryos can enter a programmed arrest of development known as diapause [75], which is accompanied by metabolic depression, a drop in the respiratory rate, as well as acidification of the intracellular milieu [74,75,76]. Such a drop in metabolic activity depends on a diminished flow of carbon into mitochondria, which is controlled by the regulatory enzymes trehalase, hexokinase, and pyruvate kinase [74]. Since trehalose is the primary fuel of *Artemia*, the activity of trehalase represents the first regulatory step in carbohydrate metabolism. Interestingly, trehalase from *Artemia* exists in two oligomeric states, which are in a pH-dependent slow equilibrium [77]. At acid pH values, a less active aggregated state of the enzyme is the predominant form, while at alkaline pH, the dissociated, more active form is the most abundant species. The transition from the aggregated state at pH 6.3 to the active species at pH 8.6 was complete in 10 min; by contrast, the reverse process requires more than an hour [77]. Thus, the transition of *Artemia* embryos from a metabolically active state into diapause is controlled, at least partially, by a hysteretic trehalase whose oligomeric equilibrium is slow and pH-dependent.

### 3.3. Hysteresis in Medicine

In mammals, blood coagulation is under strict regulation, allowing the formation of blood clots only when necessary. In this process, the activation of fibrinogen into fibrin by proteolytic cleavage represents a critical step [78]. Such activation is performed by thrombin, a serine-dependent protease [78]. In this sense, one of the early recognized features of the pathogenic bacterium *Staphylococcus aureus* is its ability to clot human blood [79]. The pathogen can trigger blood coagulation by secreting zymogen activators such as staphylocoagulase (SC) [80] and von Willebrand binding protein (VWbp) [81], which act through the formation of non-covalent complexes with prothrombin (ProT). Interestingly, the activation process is not dependent on proteolytic cleavage but instead is triggered by conformational transitions [82]. In the case of VWbp, its binding to ProT leads to the formation of an inactive ProT-VWbp complex, which is then transformed through a slow conformational change into a proteolytically active complex [83]. Such a transition is detected by a lag in the activity traces. The magnitude of the lag decreases as the concentration of VWbp increases [83]. This system represents an example of a hysteretic transition between conformational substates. The existence of hysteretic activation of the ProT-VWbp complex serves as a mechanism to avoid the premature activation of ProT. It is thought that the fibrin generated in this process helps the bacteria in evading host immune defense mechanisms [84].

The enzyme myeloblastin, also known as leukocyte proteinase 3 [85], is a serine-dependent endopeptidase present in human polymorphonuclear leukocytes [86]. The enzyme is involved in the bactericidal activity of leukocytes, which occurs at an optimum pH of 5.5. During the phagocytic process, the pH within the phagocytic vacuoles slowly decreases from 7.8 down to 5.7 [87]. Interestingly, the bactericidal activity of myeloblastin is independent of its proteolytic activity [85]. To avoid auto-digestion, purified myeloblastin is stored frozen at pH 3.2; however, the enzyme assays are carried out at pH 7. When the enzyme is diluted in the assay buffer at pH 7, the resulting progress curves reveal a lag in the enzyme activity [31]. A model accounting for an equilibrium between two conformational states of myeloblastin provides an explanation for the observed kinetic profiles [31]. At pH 3.2, the inactive conformer is the predominant form (K_eq_ = 3.75 ± 0.06). When the enzyme is added to the reaction mixture at pH 7, the equilibrium slowly changes to the active conformation, thus explaining the lag in the activity assays. It has been proposed that the hysteretic behavior of myeloblastin represents a way to delay the proteolytic activity of the enzyme during the phagocytic process [31].

## 4. Non-Hysteretic Time-Dependent Kinetic Complexities

In addition to the classical hysteretic behavior discussed above, additional time-dependent kinetic complexities have been revealed. The profile of the full progress curves of these enzymes is different when compared with that of the classical hysteretic enzymes. Such differences are magnified when the instantaneous rate profile is contrasted. Although at present restricted to specific cases, the behavior of these systems stresses the diversity of atypical kinetics that could potentially exist.

### 4.1. Damping Oscillatory Hysteresis

To date, this atypical kinetic transition has only been reported in human butyrylcholinesterase (Acyl choline acyl hydrolase EC 3.1.1.8), an acetylcholinesterase-related esterase [88]. This monomeric protein belongs to the hydrolase group of enzymes and has a broad substrate specificity, acting on molecules containing an ester bond, including acetylsalicylic acid, cocaine, succinylcholine, etc. [89]. The kinetics of the enzyme in the presence of positively charged substrates such as choline and thiocholine esters diverge from the classical Michaelis-Menten formalism [90,91]. Furthermore, time-dependent kinetic complexities have also been observed. When N-methylindoxyl acetate was used as the substrate, the progress curves of the hydrolytic reaction revealed a typical lag transition characterized by a zero initial velocity [92], suggesting the enzyme pre-exists in an inactive state. This hysteretic behavior can be explained by a simplification of the model shown in Scheme 2, assuming the initial state of the enzyme is unable to bind N-methylindoxyl acetate, leading to a slow conformational transition from a fully inactive state to one that can bind and catalyze the hydrolysis of the substrate. By contrast, with 3-(acetamido)N, N, N trimethylanilinium, a time progress curve with a burst [93] was observed.

**Figure 8:**
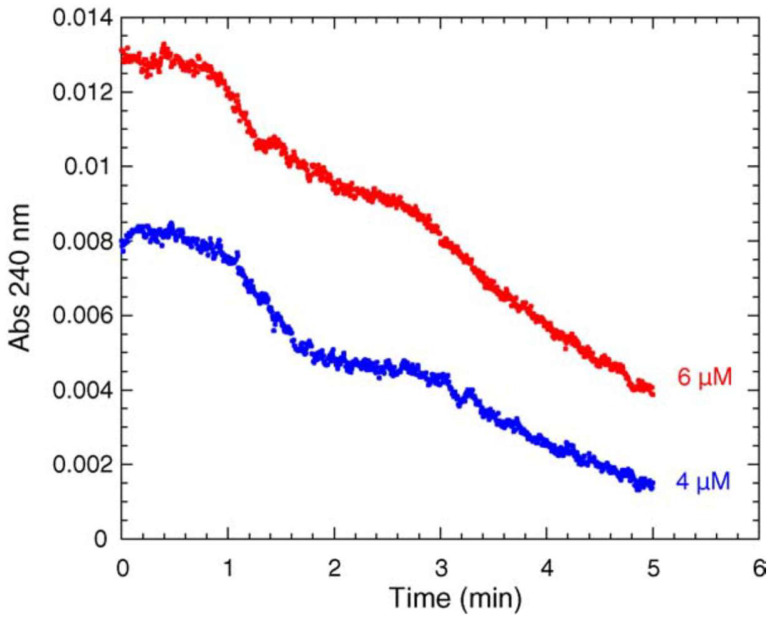
Experimental full progress curves of rat Butyrylcholinesterase with an N-alkyl derivative of benzoylcholine as substrate. Enzyme assays were carried out at 25 °C in 100 mM sodium phosphate buffer (pH 7). The enzyme concentration was 31.5 nM. Progress curves are shown as substrate consumption at either 4 µM (blue line) or 6 µM (red line). The figure was taken from reference [95] with permission.

**Figure 9:**
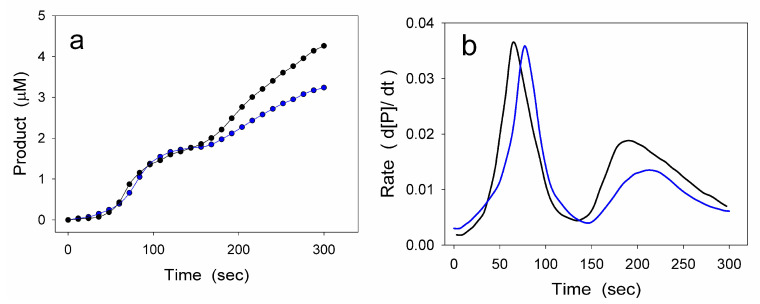
Profile of the time progress curves for an enzyme showing damped oscillatory hysteresis. (**a**) time courses of butyrylcholinesterase with an N-alkyl derivative of benzoylcholine as substrate at either 4 µM (blue dots) or 6 µM (black dots); (**b**) dependence of instantaneous reaction rate on time. Data for the elaboration of figures was taken from Figure 3 of reference [95].

However, a more complex and interesting kinetic behavior was revealed in the presence of a benzoylcholine derivative as the substrate. As shown in Figure 8 and Figure 9, the profile of the full progress curves of substrate consumption revealed oscillations that gradually dampen as the reaction progresses [94,95]. Both the frequency and the amplitude of the oscillations were shown to be a function of the substrate concentration. This atypical kinetic behavior has been described as damping oscillatory hysteresis [94,95].

To explain the observed behavior, a two-state model was proposed in which the two forms of the enzyme can bind the substrate [94]. However, only one of the two states can catalyze the hydrolysis of benzylcholine. The model’s central premise is the ability of the substrate benzylcholine to fluctuate between two conformational states, with only one state being catalytically competent. The damping oscillatory hysteresis is attributed to both the rate constants associated with the enzyme’s conformational transitions, as well as to the conversion of the substrate from the inactive to the active state. These first-order rate constants were all below 0.15 s^−1^.

**Scheme 4:**
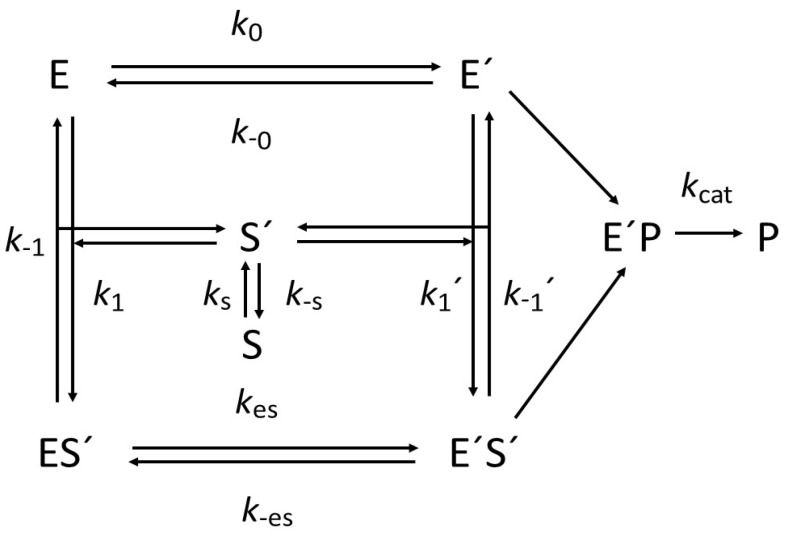
Kinetic model for an irreversible uni–uni catalyzed enzyme reaction showing a damped oscillatory hysteretic behavior. In the model, S and S’ represent different conformational states of the substrate, which are catalytically (S’) or non-catalytically competent. Only the E’S’ enzyme-substrate complex is assumed to be able to catalyze the reaction.

Scheme 4 shows the proposed model that accounts for the damping oscillatory hysteresis. As compared with the general model of hysteretic behavior (Scheme 2), the main feature of damped oscillatory hysteresis is the addition of two substates of the substrate. In the model, the rate constants *k*_1_ and *k*_1_’ are associated with substrate binding to the enzyme species E and E’, respectively, while *k*_s_ and *k*_−s_ are the first-order rate constants for the interconversion of substrate between its competent (S’) and non-competent states. As shown, only the E’S’ enzyme-substrate complex is catalytically active.

### 4.2. Instability of a Reaction Intermediary

**Scheme 5:**
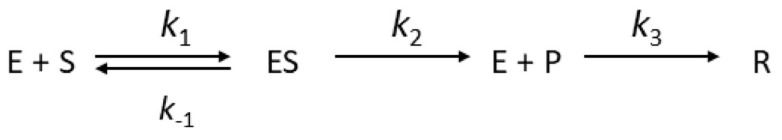
Kinetic model for an irreversible uni-uni catalyzed enzyme reaction showing an unstable reaction product.

**Figure 10:**
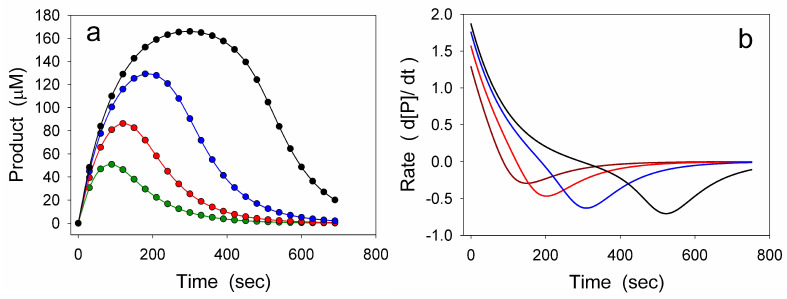
Full-time progress curves for an enzyme catalyzing a reaction in which the product of the reaction is unstable. (**a**) Full progress curves showing the effect of substrate concentration on the profile of the plots. (**b**) Instantaneous reaction rates as a function of time. Curves were generated with Dynafit software (version 4) [18] based on the model shown in Scheme 4. Values of the rate constants used in the simulation were as follows: *k*_1_ = 5 µM ^−1^ s^−1^; *k*_−1_ = 250 s^−1^; *k*_2_ = 25 s^−1^; *k*_3_ = 0.01 s^−1^. Substrate concentrations (in the micromolar scale) used in the simulations were as follows: 100 (green line); 200 (red line); 400 (blue line); 800 (black line). The initial concentration of the enzyme was 80 nM.

The product of an enzyme-catalyzed reaction is sometimes unstable, so in an enzyme assay, it is drifted into a compound with different spectral properties [96,97,98,99]. When this unstable product is used to monitor the progress of the reaction, atypical profiles of the full progress curves will be obtained (Figure 10). Similarly, an intermediary form of the enzyme generated in the catalytic cycle can also be unstable [99,100,101]. The simplest model explaining such kinetic behavior is as shown in Scheme 5:

Where R represents the compound derived from the unstable product P. The reaction has been assumed as irreversible. Unlike hysteretic enzymes, the full progress curves exhibit a maximum whose position depends on substrate concentration. Figure 10 shows simulated full progress curves (Figure 10a) and their corresponding derivative trace (Figure 10b). It is worth noting that for this kind of atypical kinetic behavior, the profile of the instantaneous rate vs. time plot (Figure 10b) will show negative values reaching a minimum in the trace. For this kind of system, no indication of atypical kinetic behavior will be revealed in the early stages of the reaction, particularly at a high substrate concentration, thus stressing the importance of analyzing the full progress curve. On the other hand, although the product accumulation plot reaches a maximum value, it is far below as compared with the corresponding initial concentration of substrate. For an irreversible reaction, such a maximum value will be a function of the magnitude of both the catalytic constant *k*_2_ and the rate constant *k*_3_ associated with the conversion of product P into the final derivative compound R.

A well-characterized example of this kinetic behavior is the oxidation of dopamine into orthoquinone catalyzed by tyrosinase (EC 1.14.18.1), a copper-containing enzyme [97]. This enzyme is involved in the biosynthesis of melanin [102]. In this case, the reaction product spontaneously evolves into an ortho-dopaminechrome by cyclization of the molecule. For those situations where the reaction exhibits reversibility, the reaction profile will not reach zero concentration of the product. It is worth noting that the profile of the full progress curves differs significantly when the reaction is monitored by following substrate consumption. Under this condition, a burst-like hysteretic profile will be observed [97].

Albeit conceptually simple, the model shown in Scheme 4 could generate complex full-time courses with maximal and minimal in the reaction profile. Such a situation will be possible when the enzyme-substrate complex is also unstable [103]. Both conditions could be present simultaneously.

### 4.3. Substrate Inhibition Followed by Product Reactivation (Kinetic Competence)

A final example of an atypical kinetic transition is represented by the multifunctional thioredoxin-glutathione reductase (TGR), an interesting splicing variant of the animal thioredoxin reductase (Thioredoxin disulfide reductase EC 1.8.1.9) [104,105,106]. The enzyme is an NADPH-dependent disulfide reductase constituted by two identical subunits [107], which is involved in the reduction of both thioredoxin and glutathione disulfide (GSSG) according to the following reactions:

Thioredoxin (S-S)+NADPH+H+ ⟶................ Thioredoxin (SH)2 + NADP+

GSSG+NADPH+H+ ⟶................ 2 GSH+NADPH+

**Figure 11:**
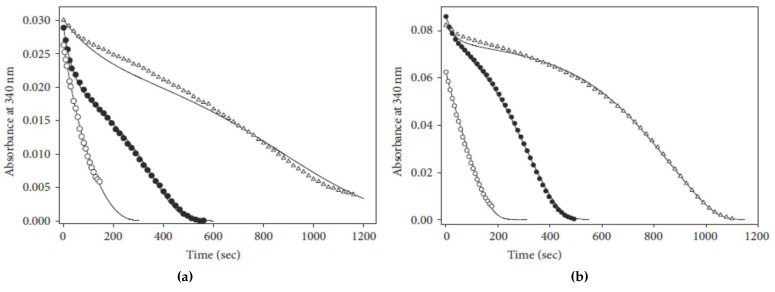
Experimental data of TGR from *Taenia crassiceps* cysticerci are shown as substrate (NAPH) consumption. Data were obtained at pH 7.8 and 25 °C at different concentrations of NADPH and GSSG to show their effect on the profile of the time progress curves. The particular conditions used were as follows: Panel (**a**) (○) 4.2 µM NADPH, 5 µM GSSG; (●) 4.7 µM NADPH, 270 µM GSSG; (∆) 4.9 µM NADPH, 500 µM GSSG. Panel (**b**) (○) 10 µM NADPH, 20 µM GSSG; (●) 14 µM NADPH, 200 µM GSSG; (∆) 13.4 µM NADPH, 510 µM GSSG. In all cases, an enzyme concentration of 11.5 nM was used. Continuous lines represent fitting of experimental data points to the mechanism-based model shown in Scheme 6. The data was taken from reference [115].

Both disulfide reductase activities are critically dependent on an essential selenocysteine residue for activity [104,108]. Each subunit of TGR is composed of four domains [105,106]: (i) an N-terminal glutaredoxin-like domain; (ii) a FAD binding domain; (iii) a NADPH binding domain, and (iv) an interface domain. The ability of the enzyme to reduce GSSG is due to the presence of the glutaredoxin-like domain appended to the N-terminal end of the typical mammalian thioredoxin reductase module [105,106,107,109]. The expression of the enzyme has been observed in a variety of mammalian tissues [110], with a particularly important role in testes [110], where it is involved in spermatid maturation. The presence of TGR is critical to flatworms, where it is the only enzyme involved in the reduction of both GSSG and oxidized thioredoxin [105,111,112,113,114]. The most interesting kinetic property of the enzyme is observed with GSSG as the substrate. At moderate or high concentrations of the disulfide, substrate inhibition is displayed. In the early stages of the reaction, the inhibition can be analyzed by the conventional steady-state kinetics [107,109,112]. However, allowing the reaction to proceed until complete depletion of NADPH reveals a variety of profiles in the full reaction progress curves [112,115]. Figure 11 shows experimental full progress curves of GSSG reductase activity by TGR from *Taenia crassiceps* cysticerci.

**Scheme 6:**
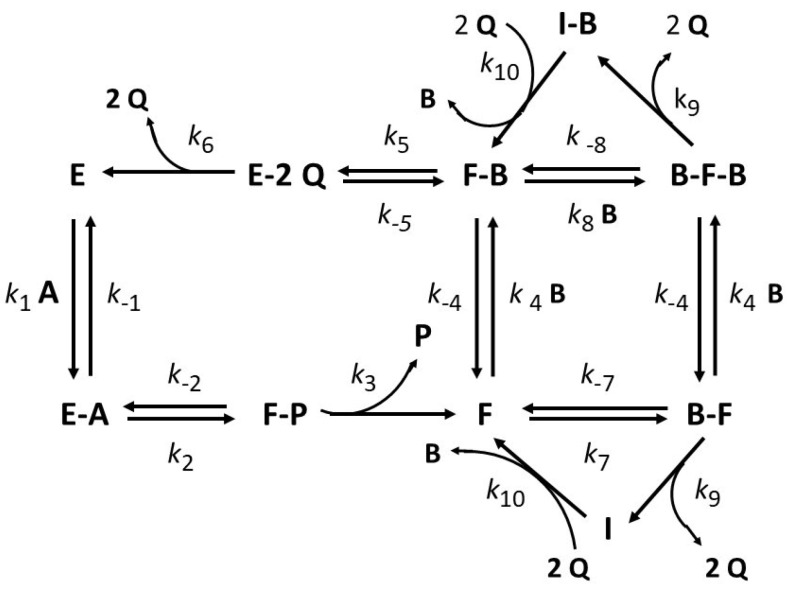
Kinetic model for an irreversible bi-bi ping pong catalyzed enzyme reaction showing a kinetic competence concomitant with reversible substrate inhibition. A and B represent the substrates NADPH and GSSG, respectively, while P and Q pertain to the products NADP^+^ and GSH. The covalent intermediary form (reduced) of the enzyme is represented by F, while I and I-B correspond to its covalently inhibited forms.

At low concentrations of both NADPH and GSSG, conventional full progress curves of substrate consumption are observed, with the slope being maximal at the start of the reaction. Increasing the GSSG concentration leads to the emergence of substrate inhibition and a complex profile in the full progress curves. Such profiles exhibit an initial burst stage followed by a lag stage at longer reaction times [115]. Interestingly, the amplitude of these phases is strongly dependent on the concentration of GSSG, NADPH, and enzyme [112,115]. At moderate or high concentrations of GSSG, the amplitude and duration of the lag stage increase, concomitant with a decrease in the amplitude of the burst stage. At high concentrations of both NADPH and GSSG, the initial burst stage was fully abolished, coinciding with the loss of enzyme activity as revealed by the initial velocity data. The order of addition of the substrates to the reaction mixture does not modify the profile of the progress curves. Interestingly, the continuous monitoring of the time course of the reaction revealed a gradual reactivation of the reductase activity, reaching an apparent steady-state segment at extended reaction times [108,112,113,114,115]. This indicates that the substrate inhibition is transient. Based on both the enzyme’s crystallographic data [106,116] and a detailed kinetic analysis [109,115], it was concluded that TGR follows a two-site ping-pong bi-bi kinetic mechanism. Further, a complex mechanism-based model was developed [115], centered on the slow binding of GSSG at an alternative second site, resulting in the formation of a glutathione-enzyme mixed disulfide, which leads to substrate inhibition (Scheme 6).

**Figure 12:**
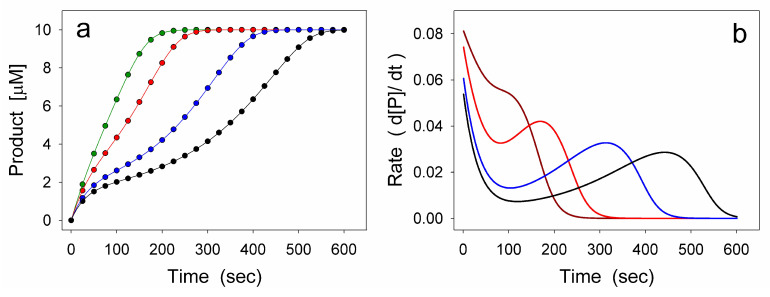
In Silico simulations of an enzyme with a ping-pong bi-bi kinetic mechanism showing a kinetic competence between two alternative pathways. (**a**) Full progress curves showing the effect of GSSG concentration on the profile of the plots. (**b**) Instantaneous reaction rates as a function of time. Data were simulated with Dynafit software (version 4) [18] based on the model shown in Scheme 6. The corresponding instantaneous reaction rate is shown in panel (**b**). The rate constants values used in the simulation were as follows: *k*_1_ = 25 µM ^−1^ s^−1^; *k*_−1_ = 480 s^−1^; *k*_2_ = 90 s^−1^; *k*_−2_ = 20 s^−1^; *k*_3_ = 160 s^−1^; *k*_4_ = 12.5 µM ^−1^ s^−1^; *k*_−4_ = 171 s^−1^; *k*_5_ = 26 s^−1^; *k*_−5_ = 22 s^−1^; *k*_6_ = 32 s^−1^; *k*_7_ = 0.075 µM ^−1^ s^−1^; *k*_−7_ = 17 s^−1^; *k*_8_ = 0.02 µM ^−1^ s^−1^; *k*_−8_ = 4.2 s^−1^; *k*_9_ = 0.06 s^−1^; *k*_10_ = 2.3 × 10 ^−4^ µM ^−1^ s^−1^. Concentrations (in the micromolar scale) of GSSG were as follows: 40 (green line); 150 (red line); 300 (blue line); 400 (black line). The initial concentration of NADPH was 10 µM. Total enzyme concentration was 30 nM.

In the model, the conventional ping-pong bi-bi catalytic cycle involves the enzyme species E, E-A, F-P, F, F-B, and E-2Q. At high concentrations of GSSG, the inhibitory cycle involving the binary B-F and ternary B-F-B enzyme complexes becomes predominant and is responsible for the inhibition observed in the enzyme assays. Due to the enzyme’s residual activity, a gradual but slow increase in the concentration of the reaction product GSH eventually reactivates the enzyme activity through the reduction in the enzyme-glutathione mixed disulfides (represented by I and I-B) [108,112,113]. The effect of adding disulfide reducing reagents (e.g., cysteine or dithiothreitol) on the atypical full progress curves is consistent with the model. Thereby, the atypical hysteretic-like progress curves of TGR are the result of a continuous competition between the conventional ping-pong bi-bi active cycle and a pathway in which substrate inhibition by GSSG is concomitant. Figure 12 shows an In Silico simulation of full progress curves represented as product (NADP^+^) formation. Clearly, the profiles match the experimental data shown in Figure 11. Interestingly, the profile of the instantaneous rate plots displays a maximum whose position is dependent on GSSG concentration.

## 5. Conclusions

The atypical kinetic phenomena discussed in the present work emphasize the importance of evaluating the full reaction progress curves for a complete kinetic characterization of an enzyme. Although in most cases the progress curves obtained in an enzyme assay will align with the expected behavior of a conventional enzyme, it is also possible to find a time-dependent complex transition. This transition could provide additional insights into the enzyme’s properties as well as a potential regulatory mechanism. Furthermore, the obtained information can elucidate the significance of the initial slope and potential late steady states. Thus, for enzymes showing hysteresis, both the initial rate at the start of the reaction and the rate at the end of the transition will give valuable information about the activities of the corresponding enzyme species present in the reaction mixture. It is worth noting that in some cases, the full progress curves may appear conventional to the non-expert worker. In such situations, the obtention of the profile of the derivative of the full progress curve (i.e., the instantaneous rate vs. time plot) and its comparison with that of a conventional enzyme will give a reliable diagnosis of the presence of a time-dependent kinetic complexity.

Additionally, an analysis through data fitting to the simplest kinetic model will reveal the presence of complex kinetic behavior. In an ideal situation, it will be advisable to analyze the kinetic behavior of the enzyme under a variety of experimental conditions (e.g., by varying pH and temperature). In the search for a model to explain the atypical behavior, detailed knowledge about the kinetic mechanism followed by the enzyme is an essential step in the analysis. In Silico simulations will also be useful in the discrimination of alternative models. In this sense, the worker must be warned about the importance of the magnitude of the rate constants involved in the model, since this will be a critical factor. A simple flow diagram to analyze an enzyme showing unexpected progress in an activity assay can be found in the Supplementary Materials section.

Finally, although the focus of the present review was centered on atypical time-dependent behavior observed in enzyme assays, such phenomena can also be found in pharmacology. At the end of the references section, readers can find some reviews dealing with such an important topic [117,118,119,120].

## Data Availability

Not applicable.

## Supplementary Materials

The following supporting information can be downloaded at https://www.mdpi.com/article/10.3390/biom15050641/s1, Figure S1: A flowchart was appended (Flowchart S1) describing the steps to analyze time-dependent complexities in enzyme assays.

## Glossary

**Hysteresis**: In enzymology, this term is defined as the delay in the response of the enzyme activity to an abrupt change in the concentration of a certain ligand, typically a substrate, to which the enzyme responds. The most common hysteretic transitions are called burst and lag. **Initial velocity**: Represents the instantaneous rate measured as either substrate consumption or product formation at the early stages of the enzyme-catalyzed reaction after the achievement of the steady-state condition. **Steady state velocity:** In enzyme kinetics, this parameter is associated with the condition under which the concentration of the productive enzyme-substrate complex remains unchanged, generating a constant rate of substrate consumption or product formation. **Burst**: In this kind of hysteretic response, the instantaneous velocity will continuously decrease from the initial velocity to the steady-state velocity. In this case, the final steady-state velocity will be smaller than the initial velocity. **Lag**: In this kind of hysteretic response, the instantaneous velocity will continuously increase from the initial velocity up to the steady state velocity. Hence, the final steady-state velocity will be higher than the initial velocity. In some specific cases of a lag hysteretic response, the initial velocity can be equal to zero. **Lag time**: Represents a measure of the time associated with the slow response to a sudden change in the concentration of a particular ligand. Its numerical value can be obtained from an experimental progress curve as the intersection point of straight lines arising from the initial and steady-state velocities. Mathematically is defined as the reciprocal of the hysteretic rate constant. Depending on the particular enzyme, its value will be in the range of seconds or minutes. **Hysteretic rate constant**: This parameter is associated with the time needed to reach the steady-state velocity in the enzyme assay. Its value will be a function of the rate constants associated with the slow transition between the conformational substates of the enzyme, the substrate concentration, and the Michaelis-Menten constants. In those cases where a slow equilibrium between different oligomeric states is present, enzyme concentration will be critical. **Amplitude:** For a hysteretic transition, it is defined as the excess in product concentration associated with a burst or the deficit in product concentration associated with a lag. Thus, for a burst kind of hysteresis, the amplitude will be positive, while for a lag kind of hysteresis, the amplitude will be negative. The magnitude of the amplitude can be estimated from the point on the ordinate axis (in concentration units) that intersects the straight line arising from the steady state velocity. **Kinetic competence**: This phenomenon occurs in situations of substrate inhibition when two catalytically competent pathways are available to the enzyme, one of which is less efficient. In an enzyme assay, kinetic competence can be observed when two alternative substrates are present, in which case two divergent catalytic pathways will be potentially attainable to the enzyme.
