# Supplementary figures and images for: Time-Dependent Kinetic Complexities in Enzyme Assays: A Review
Source: Biomolecules. 2025 Apr 30;15(5):641. doi: 10.3390/biom15050641 (PMC12108978; doi:10.3390/biom15050641)

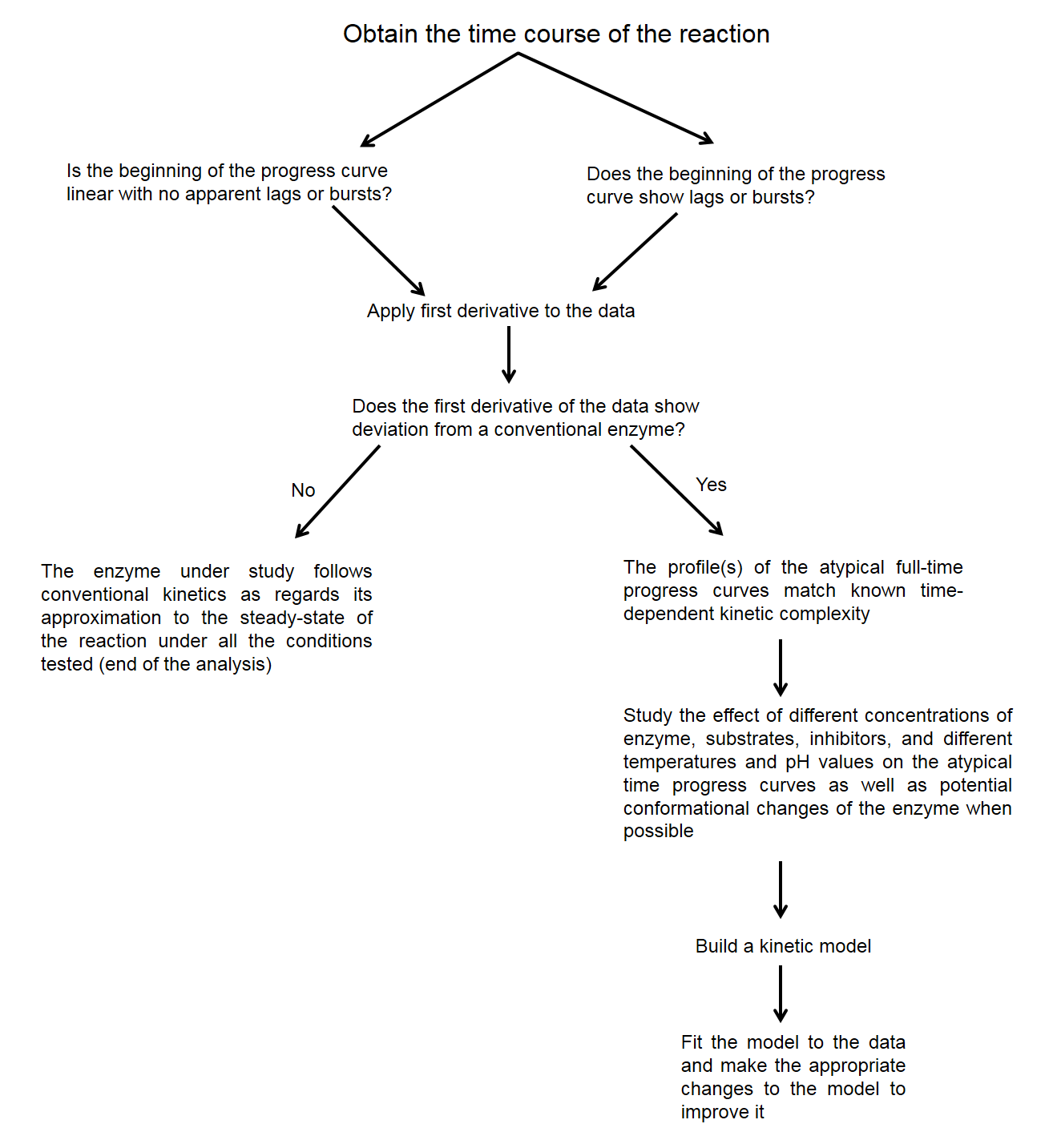

Supplement: Supplementary file 1 [file biomolecules-15-00641-s001.zip › biomolecules-3471407-supplementary.tif]
